# Paraganglioma of the Lumbar Spine: A Case Report

**DOI:** 10.7759/cureus.115849

**Published:** 2026-09-06

**Authors:** Edward Farahi-Marney, Michael Farahi-Marney, Prutha Patel, Yash Desai, Douglas Green

**Affiliations:** 1 Internal Medicine, Arkansas College of Osteopathic Medicine, Fort Smith, USA; 2 Internal Medicine, Mary Washington Healthcare, Fredericksburg, USA; 3 Internal Medicine, Edward Via College of Osteopathic Medicine, Blacksburg, USA; 4 Neurosurgery, Unity Health - White County Medical Center, Searcy, USA

**Keywords:** gross total resection, intradural extramedullary tumor, laminectomy, lumbar spine, magnetic resonance imaging, neuroendocrine tumor, paraganglioma, postoperative surveillance, rare spinal tumors, spinal cord tumor

## Abstract

Paragangliomas are rare neuroendocrine tumors, with spinal involvement representing a small minority of cases. Lumbar spinal paragangliomas are especially uncommon and may present with nonspecific symptoms, often mimicking more common intradural tumors. We describe a 49-year-old female who presented with chronic low back and sacral pain, along with bilateral lower extremity symptoms. Lumbar MRI demonstrated an intrathecal, homogeneously enhancing mass at L3 causing canal stenosis. The patient underwent lumbar laminectomy with gross total resection of an intradural, extramedullary lesion. Histopathology confirmed a paraganglioma, with immunohistochemical positivity for chromogranin A, synaptophysin, and S100. Postoperatively, she reported substantial relief of pain and radiculopathy, with no neurological deficits. Surveillance MRI at seven weeks and 4.5 months showed no residual or recurrent tumor. Although rare, lumbar paragangliomas should be considered in the differential diagnosis of intradural, extramedullary masses. Gross total surgical resection offers effective symptom relief and favorable short-term outcomes. Given the potential for delayed recurrence, we recommend close postoperative surveillance with MRI every three to six months during the initial follow-up period.

## Introduction

Paragangliomas are rare neuroendocrine tumors, morphologically similar to pheochromocytomas but occurring in extra-adrenal locations [1]. Spinal paragangliomas, particularly in the lumbar region, are exceedingly rare, often presenting in the cauda equina [2]. Most are benign and nonfunctioning, with 90% occurring as carotid body or glomus jugulare tumors [3]. The average age of presentation for spinal paragangliomas ranges from the third to fifth decade of life, with no clear sex predilection. Clinical manifestations are often nonspecific and may include sensory changes, radiculopathy, and progressive low-back pain secondary to compression of adjacent neural structures [2]. Diagnostic evaluation relies primarily on imaging, particularly abdominal and pelvic MRI. MRI characteristically identifies a well-circumscribed, highly vascular intradural-extramedullary mass with avid contrast enhancement [2,3]. Additional evaluations may include CT, functional imaging (PET or iodine-123 metaiodobenzylguanidine (I-123 MIBG) scan), and urinary or plasma evaluation for excess catecholamines [1,3]. Histopathological confirmation following surgical resection is required for a definitive diagnosis [2]. Primary treatment is centered on total surgical resection, which is associated with low recurrence rates and excellent long-term outcomes [2]. Adjuvant radiation therapy is indicated for recurrent or residual tumors, though this remains uncommon given that most lesions are benign [2]. Prognosis is generally favorable, with neurological recovery expected after adequate decompression [2]. This report describes a case of a 49-year-old female who was found to have a lumbar paraganglioma, initially identified in July 2023 and diagnosed via MRI. The case highlights key diagnostic considerations, surgical management strategies, and outcome expectations in this rare spinal neoplasm.

## Case presentation

A 49-year-old female presented with chronic, progressive low back and sacral pain, described as a “rubber band pulling” sensation in her coccyx, with bilateral lower extremity symptoms. Multiple emergency room visits and outpatient encounters occurred due to severe pain and suspected urinary tract infections. Chiropractic treatments provided no relief. Lumbar MRI revealed an ovoid, intrathecal mass at L3, filling the spinal canal and causing stenosis, without calcifications or vertebral changes (Figure 1). The patient declined non-surgical treatments and opted for surgery after reviewing risks and benefits. She underwent a lumbar laminectomy for resection of an intradural, extramedullary neoplasm in September 2023. She tolerated the procedure well, reporting improved lower extremity symptoms postoperatively. She completed antibiotics and physical therapy, was ambulatory, voiding, and tolerating a regular diet by postoperative day three, and was discharged home. At her two-week follow-up, she reported improved lumbar pain and radiculopathy. At seven weeks, postoperative MRI demonstrated no residual tumor (Figure 2), and our patient continued to endorse further symptom improvement and no neurological deficits.

**Figure 1:**
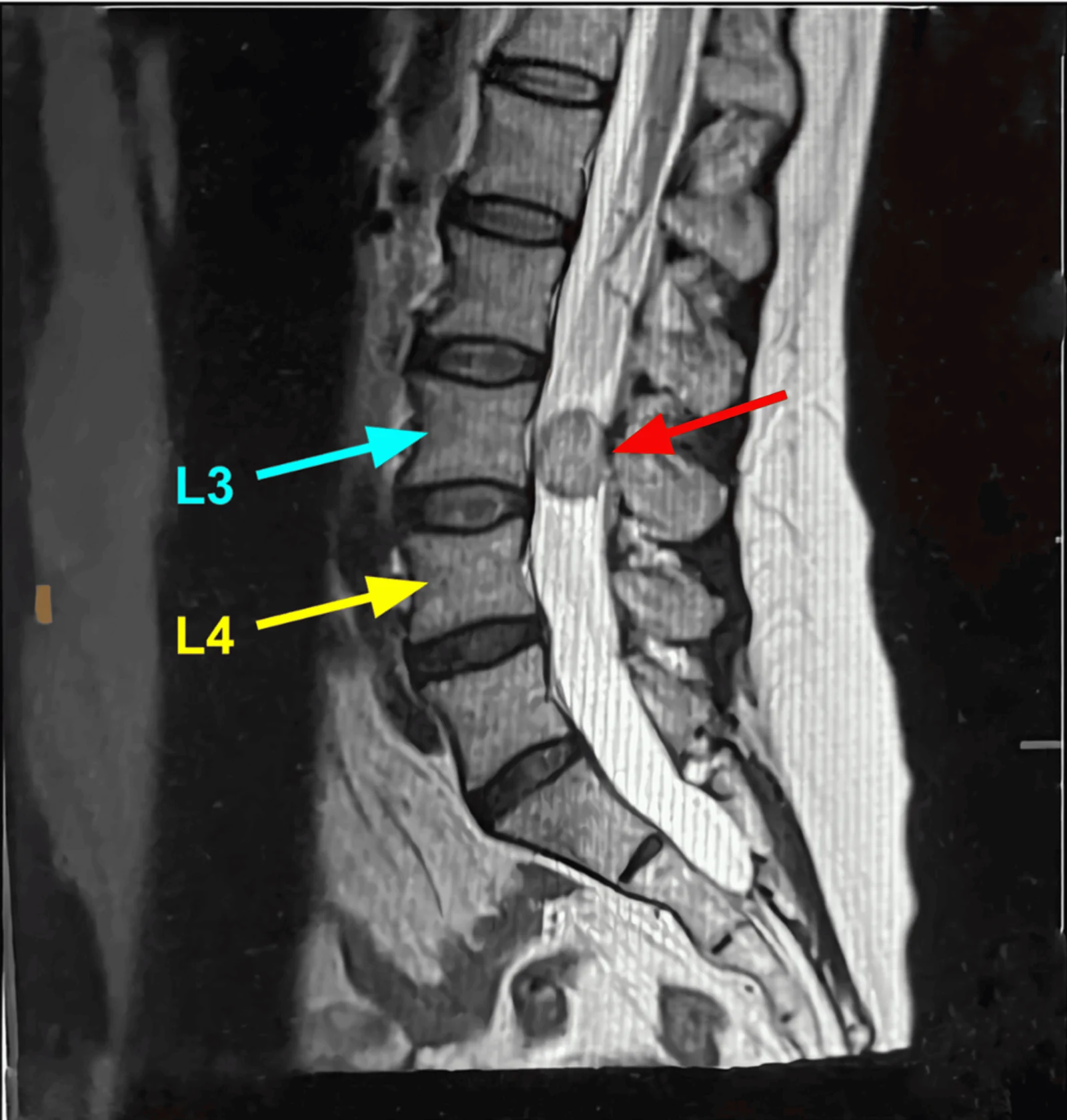
Sagittal T2-weighted MRI of the lumbosacral spine demonstrating the preoperative L3 intrathecal mass causing canal stenosis. Dominant abnormalities involve L3, where an ovoid, intrathecal mass fills the spinal canal (red arrow).

**Figure 2:**
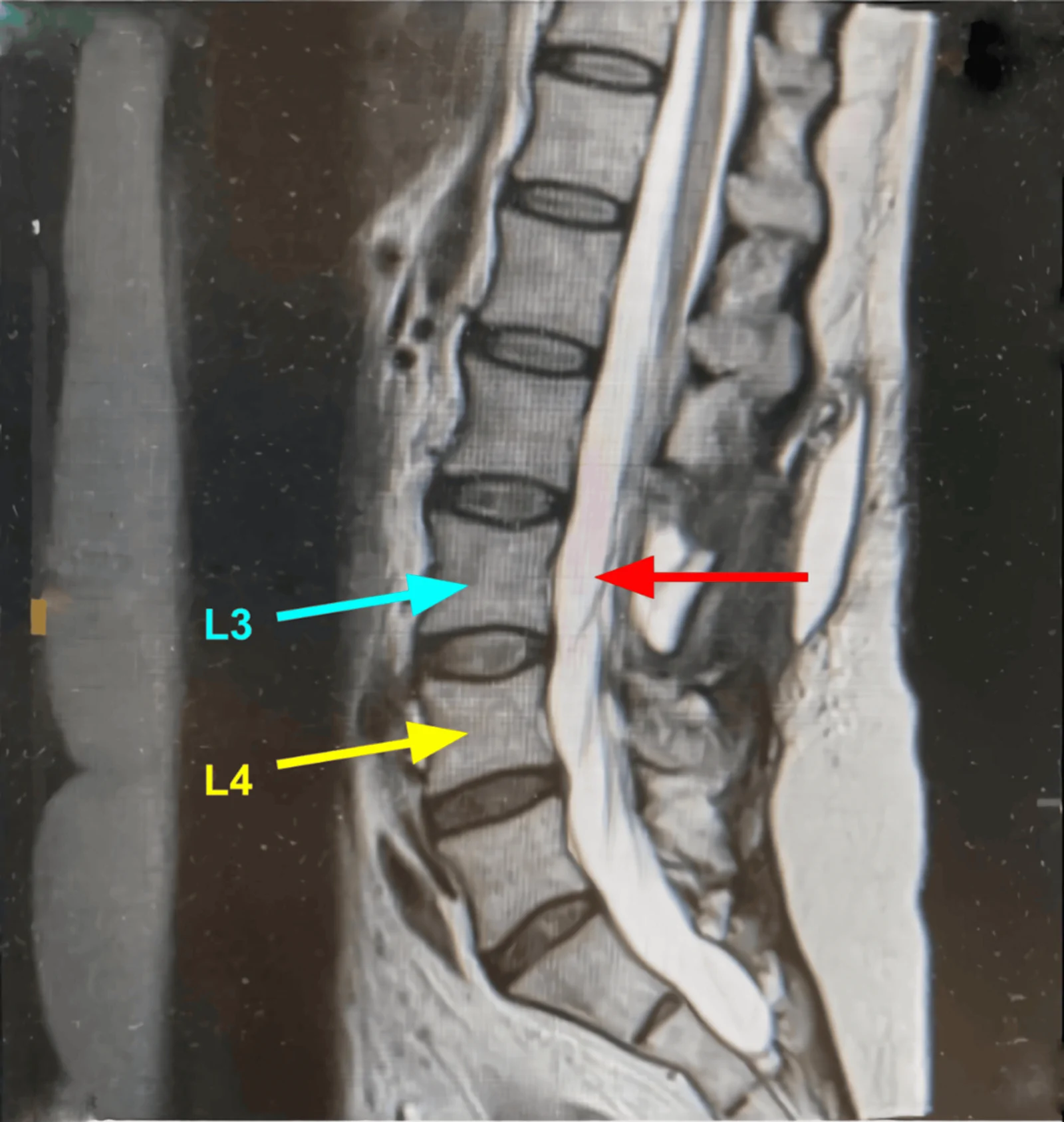
Sagittal T2-weighted MRI of the lumbosacral spine taken seven weeks after surgery with no signs of residual tumor (red arrow).

Lumbar laminectomy was performed for resection of an intradural, extramedullary neoplasm at L3, indicated by chronic low back/sacral pain and bilateral lower extremity symptoms. Preoperative diagnosis was a neoplasm of uncertain behavior. After consent, the patient was placed under general anesthesia in the prone position with chest and pelvic bolsters. Intraoperative fluoroscopy localized the L3-L4 region. A midline incision was made, and bilateral subperiosteal dissection exposed the L3 and L4 laminae. Laminectomy was performed using Leksell and Kerrison rongeurs, resecting the ligamentum flavum. A midline durotomy was made, and a vascular ovoid mass was identified intradurally. Microdissection freed the mass from the dura and nerve roots, with vascular pedicles coagulated and divided. Frozen section suggested a benign vascular tumor. Gross total resection was achieved, and the dura was closed with 4-0 Surgilon (Medtronic, Dublin, Ireland), with no cerebrospinal fluid leakage. The wound was irrigated and closed in layers.

The resected 2.1 × 2.0 × 2.2 cm soft, tan nodule showed the following immunohistochemical findings: pancytokeratin, weakly positive; S100 protein, positive; chromogranin A, positive; and synaptophysin, positive. These findings confirmed a paraganglioma, with positive chromogranin A and synaptophysin on immunohistochemistry indicating neuroendocrine differentiation, and S100 suggesting sustentacular cells. Corresponding immunohistochemical images were unavailable for inclusion in this report; the findings described above are based on the original pathology report.

This case highlights the successful recovery of a 49-year-old female with a rare lumbar paraganglioma causing chronic low back pain, sacral discomfort, and bilateral lower extremity symptoms. MRI revealed an L3 intrathecal mass causing canal stenosis. After declining conservative treatments, she underwent a lumbar laminectomy with gross total resection, confirmed as a paraganglioma. Postoperatively, she reported significant symptom improvement, with no recurrence at four and a half months, underscoring the efficacy of surgical intervention.

## Discussion

The patient’s symptoms, including suspected bladder dysfunction, suggested potential cauda equina compression due to the L3 tumor’s size and canal stenosis [4]. MRI was pivotal in characterizing the intradural extramedullary lesion and narrowing the differential diagnosis [5]. The differential diagnosis included meningioma, schwannoma, and ependymoma [6], but pathology confirmed paraganglioma. The absence of intraoperative neurophysiological monitoring, unlike some reports [7], did not compromise outcomes, as gross total resection was achieved.

Spinal paragangliomas are rare, constituting 2-8% of paragangliomas, with an incidence of 0.3-2.0 per 100,000 [8]. Surgical resection is the standard of care, achieving symptomatic improvement in 86.2% of patients and a 3.9% recurrence rate [9]. Cases without bone invasion show no recurrence or metastasis [10]. Neurological deficits often improve post surgery, though motor and sphincter dysfunction recover slowly [11]. Recurrence rates vary, with primary cases showing lower risk than metastatic ones [12]. Adjuvant radiotherapy may control tumor growth in incomplete resection cases [13], but its efficacy remains uncertain [14]. Chemotherapy is reserved for malignant cases [15]. Diagnosis is challenging due to nonspecific features, often requiring histological confirmation [16].

The procedure used intraoperative fluoroscopy for precise localization and microdissection to preserve nerve roots, addressing the tumor’s vascularity. No intraoperative monitoring was used, yet outcomes were excellent [7]. This tailored approach, combined with a cautious follow-up plan (imaging every three months initially), reflects the tumor’s rarity and recurrence risk.

Postoperative care included antibiotics and physical therapy. The patient was ambulatory and discharged on day three. At two weeks, she reported improved pain and radiculopathy. An MRI at seven weeks showed no recurrence (Figure 2), with 5/5 muscle strength and normal gait. At four and a half months, she returned to work, with no recurrence on MRI. Long-term clinical and imaging surveillance is reasonable given the possibility of delayed recurrence.

This case aligns with literature reporting high success rates for intradural tumor resection. Li et al. noted 96.3% total resection in 27 neurilemmoma cases with significant improvement [17]. Cofano et al. reported 84.3% gross total resection in 249 cases [7]. Minimally invasive approaches may reduce complications [18], suggesting future consideration.

Surgical resection is effective for lumbar paragangliomas, with this case demonstrating complete symptom relief. The L3 location posed cauda equina compression risks, mitigated by prompt surgery [4]. Frequent imaging (every three to six months initially) is recommended due to recurrence risks, particularly with SDHB mutations [19]. Future studies should explore long-term outcomes and minimally invasive techniques [7,18].

## Conclusions

This case highlights that lumbar paragangliomas, although exceptionally uncommon, can be effectively managed with early recognition and gross total resection. The patient’s chronic low back pain, sacral discomfort, and bilateral lower extremity symptoms improved following lumbar laminectomy for resection of an intradural, extramedullary mass at L3. Follow-up imaging at four and a half months demonstrated no recurrence. Given the tumor’s potential for late recurrence, frequent postoperative imaging is recommended. Continued reporting of rare spinal paragangliomas is essential to inform timely surgical management and long-term care strategies.
